# Development of foraging skills in two orangutan populations: needing to learn or needing to grow?

**DOI:** 10.1186/s12983-016-0178-5

**Published:** 2016-09-29

**Authors:** Caroline Schuppli, Sofia I. F. Forss, Ellen J. M. Meulman, Nicole Zweifel, Kevin C. Lee, Evasari Rukmana, Erin R. Vogel, Maria A. van Noordwijk, Carel P. van Schaik

**Affiliations:** 1 Department of Anthropology, University of Zürich, Winterthurerstrasse 190, 8057 Zürich, Switzerland; 2 Fakultas Biologi, Universitas Nasional, Jl. Sawo Manila, RT.14/RW.3, Ps. Minggu, DKI Jakarta, Indonesia; 3 Department of Anthropology, Rutgers, The State University of New Jersey, New Brunswick, NJ 08904 USA

**Keywords:** Body growth, Development, Diet repertoire, Feeding rates, Foraging skills, Life history, Needing-to-learn hypothesis, Ranging, Orangutans, Skill learning

## Abstract

**Background:**

Orangutans have one of the slowest-paced life histories of all mammals. Whereas life-history theory suggests that the time to reach adulthood is constrained by the time needed to reach adult body size, the needing-to-learn hypothesis instead suggests that it is limited by the time needed to acquire adult-level skills.

To test between these two hypotheses, we compared the development of foraging skills and growth trajectories of immature wild orangutans in two populations: at Tuanan (*Pongo pygmaeus wurmbii*), Borneo, and Suaq Balimbing (*Pongo abelii*), Sumatra. We collected behavioral data on diet repertoire, feeding rates and ranging competence during focal follows, and estimated growth through non-invasive laser photogrammetry.

**Results:**

We found that adult-like diet repertoires are attained around the age of weaning and that female immatures increase their repertoire size faster than their male peers. Adult-level feeding rates of easy techniques are reached just after weaning, but several years later for more difficult techniques, albeit always before adulthood (i.e. age at first reproduction). Independent immatures had faster feeding rates for easy to process items than their mothers, with male immatures achieving faster feeding rates earlier in development relative to females. Sumatran immatures reach adult-level feeding rates 2–3 years later than their Bornean peers, in line with their higher dietary complexity and later weaning. The range-use competence of independently ranging and weaned immatures is similar to that of adult females. Body size measurements showed, immatures grow until female age of first reproduction.

**Conclusions:**

In conclusion, unlike in humans, orangutan foraging skills are in place prior to reproduction. Growth trajectories suggest that energetic constraints, rather than skills, best explain the length of immaturity. However, skill competence for dietary independence is reached later where the adult niche is more complex, which is consistent with the relatively later weaning age with increasing brain size found generally in primates, and apes in particular.

**Electronic supplementary material:**

The online version of this article (doi:10.1186/s12983-016-0178-5) contains supplementary material, which is available to authorized users.

## Background

Compared with other primates, great apes have an exceptionally long period of development, even after controlling for the effects of body size [1, 2]. Among them, with a developmental period of almost 20 years, humans are the most extreme case [3]. So far, the reasons for this delay of the reproductive period remain poorly understood. According to classic life history theory, although the developmental period is needed to grow both the body and brain to adult size, the reproductive phase should be reached as early as possible to maximize reproductive outcomes. Thus, the relative length of the immature phase is determined by the optimization of developmental and reproductive schedules in the face of allocation tradeoffs and externally imposed mortality [1, 3–5]. However, variation in the length of the immature phase cannot be explained completely by these tradeoffs, particularly among great ape species.

Much of this remaining variance has been explained by brain size. When controlling for body size, larger-brained species have a relatively longer period of development than smaller brained species [3, 6, 7]. The prolonged immature period of the large-brained great apes is largely in line with this. However, the question remains why such a correlation exists in the first place. Two main theoretical frameworks have been proposed to explain the correlated evolution between the length of the developmental period and brain size.

First, it has been suggested that larger-brained animals show an extended developmental period because they need time to acquire more and increasingly complex skills required for adult survival and reproduction (*needing*-*to*-*learn hypothesis*: [8]). In this view, the length of the developmental time is constrained by the number and complexity of skills a species must acquire. This hypothesis therefore predicts that at least some vital skills needed for adult survival reach adult levels just before the age at first reproduction. This prediction holds for birds, whose adult-level foraging skills are attained around the time of first reproduction [9–11]. In most mammals, however, skill competence is reached well before the age at first reproduction [11, 12]. In contrast to birds, most mammals need to be self-supporting right after weaning due to the lack of post-weaning provisioning [13]. Since the consequences of food acquisition incompetence are especially severe during development [12], at weaning a juvenile’s skills must have reached a level sufficient to support their smaller but still growing body. It is thus likely that for most mammals, skill acquisition may determine the timing of weaning, even though it does not directly constrain the age of first reproduction [3].

The competing hypothesis is that energetic constraints of large brains on somatic development prolong the length of the developmental period (e*xpensive brain framework*: [14, 15]). This framework encompasses several hypotheses proposed earlier, including the effects of maturational constraints and malnutrition avoidance [16]. Brain tissue is among the most energetically expensive tissues to maintain [17] and requires even more energy to develop [18]. Also, because developing brains are especially susceptible to temporary energy shortages [19], brain development must be conservative so it can be constantly supplied by sufficient energy. Brain growth is completed before somatic growth [20, 21]. Accordingly, the high energetic investment of larger-brained species into brain development during infancy and juvenility results in a delay in the physical development of the body, and thus a delayed onset of the reproductive period [14, 16, 22]. The *expensive brain framework* is in line with the interspecific correlation between large brains and delayed maturation. Within species, it predicts that body growth is only completed around the age of fist reproduction, particularly in females.

A comparative study has shown that in most mammals, foraging skills are in place well before the age of first reproduction and thus unlikely to be limiting the onset of reproduction [11]. Data on chimpanzees (*Pan troglodytes*) and gorillas (*Gorilla gorilla beringei and Gorilla gorilla gorilla*) suggest that adult-like diet repertoires and feeding rates are reached around the age of weaning, with the exception of chimpanzee tool use and cooperative hunting, for which competence is reached well after weaning [23, 24]. Most other primate species seem to follow this pattern of all major foraging skills being reached by weaning, whereas top-end-complexity techniques without systematic dependence (e.g. tool use) may be reached later. Thus, competence in all foraging skills roughly coincides with weaning in chacma baboons (*Papio ursinus*, [25]), bonnet macaques (*Macaca radiata*, [26]), common marmosets (*Callithrix jacchus*, [27]), squirrel monkeys (*Saimiri sciureus*, [28]) and Mayotte brown lemurs (*Eulemur fulfus*, [29]). In very few primate species, foraging competence is reached significantly later than weaning, such as in brown capuchins (*Cebus apella*, [30, 31]), or even only around the age at first reproduction as in Japanese macaques (*Macaca fuscata*, [32, 33]) and hamadryas baboons (*Papio hamadryas*, [34]). However, in the latter cases, lower foraging performance can arguably be attributed to a lack of strength (due to smaller body size of the immatures) rather than a lack of skills. Overall, this pattern suggests that in primates, the need to acquire sufficient foraging skills determines the age of dietary independence rather than the age of first reproduction.

Of all primate species, orangutans have the most extreme life history. They have the latest age at first reproduction of any nonhuman primate species and the latest age of weaning and interbirth intervals of any primate species [35]. In line with these extremely slow rates of growth and reproduction, orangutans have the lowest total energy expenditure of all great apes [36]. Furthermore, there is some variation in these life-history parameters between the different orangutan species and subspecies. Immature Sumatran orangutans are weaned around the age of 7.5–9 years, which is 1–2 years later than their Bornean peers [37]. Weaning is followed by a multiyear juvenile period during which individuals are fully self-supporting (see below) but not yet reproducing. Whereas Bornean orangutan females have their first offspring around the age of 13–14 years, their Sumatran orangutans wait for another 2–3 years until they start reproducing, around the age of 14–16 years [37–39].

At the same time, compared with other great apes, orangutans have a rather solitary lifestyle, with the degree of sociability ranging from semi-solitary to low level fission-fusion, depending on populations [40–43]. After having been in constant and close associations with their mothers during infancy, juvenile orangutans start to range more independently within 1–2 years of completing weaning [37, 44]. Depending on species and population (Sumatran orangutans tend to be more sociable than Borneans), independent immatures spend 30–80 % of their time on their own, whereas for the remaining time they mainly associate in small peer groups [37, 44, 45]. Unlike most other primates, therefore, orangutans begin to range primarily alone soon after weaning, and thus cannot systematically rely on social information for any of the skills they depend on (see below). It is therefore possible that the late weaning of orangutans, relative to other primates, is linked to the need to sustain themselves independently soon afterwards.

The aim of this paper is to investigate whether the exceptionally late age at weaning and first reproduction of wild orangutans (*Pongo* spp.) is best explained by the time needed to develop adult-levels skills or by the energetic constraints imposed from competition for energy between brain and body growth and differentiation. Given their lack of regular coalitions or other complex social interactions, social skills are unlikely to be a constraining factor on orangutan development. For all non-food subsistence skills (e.g., nest building) competence is already reached during infancy [44]. Thus, probably the most crucial skills immature orangutans have to acquire are foraging skills. Orangutans live in a complex foraging niche: food availability in most orangutan habitats fluctuates during and across years without following any clear seasonal pattern [46]. They have very broad diets and rely on a variety of difficult to process food items. Some populations habitually use tools in the foraging context [47–49]. Foraging skills can be divided into food selection competence (what to eat), food processing competence (how to eat), and food locating competence (where and when to eat; [50]). Given their broad diets, complex processing techniques, and highly fluctuating food availability, each of these three aspects may limit orangutan skill development and their ability to compete in the adult niche. For any foraging skills to limit development, they must be learned rather than innate. Indeed, it takes immature orangutans multiple years to acquire their foraging skills. Also, there is evidence that they do so by a combination of social- and individual learning [37, 51, 52].

We first test the predictions of the *needing*-*to*-*learn hypothesis*. These are that full adult competence is reached (i) around the age at first reproduction if skill levels limit reproduction or (ii) around weaning if skill levels limit the age of nutritional independence. For this we will follow Rapaports and Brown’s [50] functional division of foraging skills by looking at the development of diet repertoires (*what to eat*), feeding rates (*how to eat*), and ramble ratios of the travel routes, i.e. the actual path length divided by distance between beginning and end of daily path (*when and where to eat*). The *needing*-*to*-*learn hypothesis* further predicts that (iii) if foraging skills are learned, more complex skills are attained later than less complex skills, and that (iv) life history differences between the populations will be reflected in skill trajectories and dietary complexity of the population, such as that later age at first reproduction or later weaning should go in hand with a later age of skill competence and a more complex diet. Finally, since male and female orangutans have different energetic needs and time budgets [53], it is possible that females focus more on the development of ecological skills compared to males, and thus that we will find sex differences in acquisition trajectories.

To test the predictions of the *expensive brain framework*, we will estimate growth trajectories of the immatures in the two populations. If, as under the assumptions of the *expensive brain framework*, energetic constraints are limiting the age at first reproduction, we predict that immature females should continue growing until at least the age of first reproduction.

## Methods

### Data collection

Data were collected at Suaq Balimbing (3°42′N, 97°26′E, Aceh Selatan, Indonesia) and at Tuanan (2°09′S, 114°26′E, Kalimantan Tengah, Indonesia), on a population of wild Sumatran (*Pongo abelii*) and Bornean orangutans (*Pongo pygmaeus wurmbii*), respectively. Both study sites consist mainly of peat swamp forest and have high orangutan densities (7 individuals per km^2^ at Suaq and 4 at Tuanan, [54]). Only at Suaq do the orangutans use tools in the foraging context. Tool use can be divided into two broad classes: insect tool use, where sticks are used to get access to insects or their products in tree holes, nests and other substrates, and fruit tool use, where sticks are inserted into the valves of the fruits of *Neesia aquatica* to facilitate seed extraction [47, 49].

To assess skill development we examined 13 immature individuals from Suaq and Tuanan each, using their own mothers as adult references. Details on the focal individuals are summarized in the appendix (Additional file 1: Table S1). Data availability varies for each of the analyzed aspects and thus, so do sample sizes across the different analyses. Which data were used for each analysis is summarized in Additional file 1: Table S1. We distinguish two categories of immatures: dependent immatures (infants), who are between birth and weaning; and independent immatures (juveniles), who are between weaning and female age at first reproduction. Weaning age at Suaq is around 7–9 years and at Tuanan around 6–7.5 years, whereas female age at first reproduction is around 13–14 years at Tuanan and 15 – 16 years at Suaq ([39], and van Noordwijk and Schuppli unpublished data).

#### Diet repertoires

Diet repertoires were assessed with the help of long-term behavioral databases compiled at the two field sites. At Tuanan, data have been collected since 2003 and at the time of analysis the database contained 33′200 follow hours on adult females and immatures. At Suaq, data have been collected since 2007, and around 8′400 h of data have been collected on adult females and immatures. These data were collected during focal follows, following the same protocol for orangutan data collection (http://www.aim.uzh.ch/de/research/orangutannetwork/sfm.html), using focal animal sampling including instantaneous scan sampling at two minutes intervals. Inter-observer reliability was assessed based on simultaneous follows by multiple observers on the same focal animal without verbal exchange about the activity of the focal animal. New observers had to reach an index of concordance of at least 85 % with experienced observers before their data was included in the database.

When the focal animal was feeding, we recorded the species as well as the food item. For the analysis, we used the broad categories of fruits, leaves (and other green vegetative matter in the case of vines and lianas), inner-bark, pith and insects. Each species – part combination was considered as one specific “food item”. Adult females at Tuanan each have diets comprising around 170 or more food items from around 110 plant species. It took more than 1500 follow hours collected over multiple years before the recorded repertoire sizes of these females started to stabilize (see Additional file 2: Figure S1) and even after 4000 follow hours, new items are still being recorded. Thus, the development of diet repertoires could only be analyzed at Tuanan where the database is currently far more extensive than at Suaq.

We examined the development of the diet repertoire in 6 immatures at Tuanan (3 females and 3 males) on which we had multiple years of continuous and dense data (at least 400 follow hours for each year of data collection) covering 4.5–7.5 years of their infancy (for details on the used data set, see Additional file 1: Table S1). The overlap with the mother was calculated for continuous consecutive blocks of 450–500 follow hours, which correspond to 9–13 months each. Since for each data point the overlap calculations are based on data simultaneously taken on the offspring and its mother (followed on the same days), they are based on exactly the same amount of hours for both mother and offspring. However, to correct for the fact that older infants have been followed longer and are compared to a longer follow period of their mothers, we included follow effort as a factor in the statistical model.

#### Feeding rates

Feeding rates were collected directly during focal follows by CS from 2010–2015 as well as from close-up videos of the focal animals feeding, taken by various observers in 2012–2015. We calculated how much time it took an individual to process a food item from picking to ingestion. In the field this was accomplished using a stopwatch. The videos were coded using the interact software (Interact 9). Feeding rates that were taken directly in the field did not differ from those obtained by video coding on the same food item by the same adult focal animal (Additional file 1: Table S2). The complexity of the food items was determined by the number of steps it takes to process an item (for details see Table 1).

**Table 1:** Processing steps of food items: the most frequent combinations of the different processing steps, as well as descriptions of the corresponding food items with local and scientific names of example species (T = Tuanan, S = Suaq)

Nr	Processing steps	Food item types	Examples
0	Pick	Fruits and flowers where everything is eaten	Lunuk (*Ficus sp*.; T), Tapuhut Putih (*Syzigium sp*.; T), Nyatoh Puntik (*Palaquium pseudorostrum*; T), Resak Ubar (*Brackenridgea palustris Bartell*; S), Tapis Batu (*Garcinia sp*.; S)
1	a) Pick, bite off	a) Fruits and flowers where a small outer part is bitten off after picking, discarded, and the remaining parts are eaten	a) Medang Baru (*Litsea gracilipes Hook f*.; S), Nyatoh undus buah merah (*Palaquium ridleyi*; T), Katiau (*Madhuca motleyana*; T), Mangkinang Blawau (*Elaeocarpus sp*.; T)
	b) Pick, drop	b) Fruits and flowers where only the sap is ingested and all other parts are discarded after chewing	b) Rewui (*Microcos sp*.; T), Piais (*Nephelium mangayi*; T), Tampang (*Artocarpus dadak*; T)
2	a) Pick, peel, spit out	a) Fruits where the pulp is eaten while the skin and the seed are discarded	a) Puwin (*Sandoricum beccarianum Baill*.; S), Papung (*Sandoricum borneense*; T)
	b) Pick, bite in half, scrape flesh out	b) Hard-shell fruits where pulp and seeds are eaten but the empty pod is discarded	b)Malaka (*Tetramerista glabra*; S), Lewang (*Pouteria cf malaccensis*; T)
	c) Pick, turn repeatedly in mouth, drop seed and skin layers	c) Fruits with edible flesh tightly attached to an inedible seed and thin skin	c) Enyak Beruk (*Syzygium sp*.; T), Nyatoh undus buah besar (*Palaquium cochlearifolium*; T), Tantimun unripe (*Tetrameristra glabra*; T)
	d) Pick, pop pod open, extract seed	d) Fruit pods with an edible seed enclosed	d) Ubar (*Horsfieldia crassifolia*; S), Akar Kamunda (*Leucomphalos callicarpus*; T)
3	a) Pick, bite in half, scrape flesh out, spit out	a) Hard shell fruits where the pulp and seeds are eaten but the empty pod and seeds are discarded	a) Malaka unripe (*Tetramerista glabra*; S), Karandau Putih (*Blumeodendron kurzii*; T), Tutup Kabali (Diospyros pseudo-malabarica; T)
	b) Pick, peel, bite away flesh, remove skin around seed	b) Fruits where the skin around the seed is eaten after removing the inedible skin and flesh	b) Manga Hutan (*Mangifera gracilipes Hook f*.; S)
	c) Pick, pop pod open, extract seed, pop seed open	c) Fruit pods with a seed enclosed; only the internal part of the seed is eaten while the rest is discarded	c) Sepang (*Sterculia sp*.; S)
	d) Collect substrate, scratch or bite open, suck	d) Insects embedded in wood or other substrate	d) Ants (Formicidae; T + S), Termites (*Isoptera*; T + S)
	e) Bite piece of bark loose, rip or strip it off, scrape inner bark off	e) Inner bark (i.e. i.e., cambium/phloem)	e) Maruang (*Myristica lowiana*; T), Pantung (*Dyera lowii*; T), Resak Payo (*Dialium patens Backer*.; S)
4	a) Pick, bite tip off, pull string off of pod to open it, turn pod open, extract seed	a) Bean-like fruits with inedible skin but edible seeds	a) Basong (*Alstonia spatulata BI*.; S)
	b) Pick, pop pod open, extract seed, pop seed open, extract and spit out skin around seed	b) Fruit pods with a seed enclosed; only the internal part of the seed is eaten while the inedible seed skin is discarded	b) Sepang unripe (*Sterculia sp*.; S)
	c) Break dead twig off, examine, bite appart, suck	c) Ants hidden in hollow twigs	c) Ants (Formicidae; S)
5	a + b) Break stick off, peel (optional), chew (optional), insert into tree hole/ nest extract insects or insect product, collect from tool tip	Tool use: a) tree hole b) insect nests	a) Sweat bees (Stingless bees: *Halictidae spp*.; S) b) Ants (Formicidae; S), Termites (*Isoptera*; S), Bees (*Apidae spp*.; S)
	c) Break fruit off, break stick off, peel (optional), chew (optional), insert into tree fruit, extract seed, collect from tool tip, spit out seed skin	c) fruits	c) Cemenang (*Neesia aquatica*; S)

Feeding rates were collected for 11 and 10 immature individuals and their mothers at Tuanan and Suaq, respectively. Rates were averaged over a minimum of five individual samples (of the same individual feeding on the same food item). To capture the development of feeding rates with age, if there was a gap of more than 5 months between two groups of measurements of the same individual feeding on the same species, these measurements were treated as a different data points. To account for the fact that we thus had multiple data point of the same individual feeding on the same food item (at different stages of its development), we analyzed our data using mixed models (see below). To avoid the possible confounding effect of different stages of ripeness of the fruits on feeding rate, whenever possible (60 % of all cases), feeding rates of the immatures were calculated as a percentage of their mothers’ feeding rates when feeding in the same tree at the same time. However, in the remaining 40 % of the cases (mainly for older, independently ranging immatures), the mother’s data were not available and thus the average feeding rate of the adult female feeding on the same food item closest to the measurement date of the immature was taken as an adult reference. The presence or absence of simultaneous samples of the mother had no effect when we included it as a binary variable in the model (see Additional file 1: Table S3).

#### Ranging competence

We assessed ranging competence by estimating ramble ratios (sinuosity values) of the travel routes, assuming that individuals that struggle to plan their daily resource exploitation schedule would show higher ramble ratios. For this, locations of the focal animals were taken every 30 min during focal follows using GPS devices (Garmin models GPSMAP 62 s, GPSMAP 60CSx, and GPSMAP 78). Ramble ratios were then calculated in Arc GIS [55], using Hawth’s tool [56] and represent the total path length divided by the distance between start and end point of the follow (nest locations for full day follows). Average daily ramble ratios were calculated based on 5 – 17 ($\overline{\mathrm{X}}$ = 9) follow days within the same 5 months, to capture one specific developmental state of the juvenile individuals, which resulted in a total of 17 age/individual data points of 8 different juvenile individuals (see Additional file 1: Table S1 for more details). To ensure maximal comparability (e.g. in terms of possible seasonality effects), as adult references, ramble ratios of the mothers followed during the same periods (within the same 5 months) were used. Because this often resulted in >1 data points per individual, we used a mixed model approach (see below).

#### Body size measurements

For body size measurements, we relied on laser photogrammetry [57, 58] using a camera (Nikon D 90) with 3 parallel lasers attached at a constant distance. The laser points visible in the pictures were used as a reference scale bar. The camera was calibrated by taking pictures of a known reference scale at various distances in the forest before the actual measurements. We focused on three measures of arm length: forearm (elbow to wrist), upper arm (shoulder joint to elbow) and whole arm (shoulder joint to wrist) as a proxy for overall body size.

**Fig. 1:**
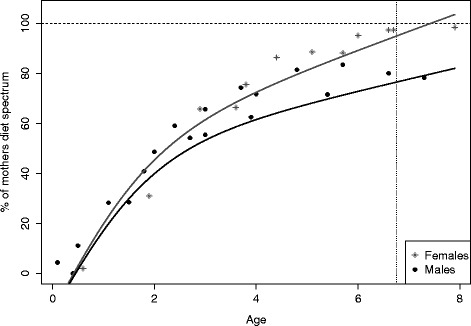
Development of diet repertoire at Tuanan: Diet repertoire size in percentage of the mother’s diet repertoire size in relation to age (in years) for immatures that are still in permanent associations with their mothers. The dotted vertical line shows mean weaning age at the Tuanan population. The dashed horizontal line marks the mothers’ diet repertoire sizes (100 %)

Laser pictures were taken at Suaq Balimbing in 2013–2014 by various observers. Distances in the pictures were calculated by KL using Paint.net Software. Data points represent averages of 4 – 22 ($\overline{\mathrm{X}}$ = 9.1) measurements of the same individual taken within the same 5-month-period. Only pictures showing the arms perpendicular to the field of view of the camera were used. The results presented here are preliminary because only a handful of individuals were available and the standard deviations of the measurements were also still high (see Fig. 1 a). The data from Suaq was compared to data on the Tuanan immatures taken by Abigail Philips in 2009–2010 on the same focal individuals as observed in the current study and using the same measurement technique and camera set up [58].

#### Dietary complexity

We assessed the dietary complexity of the four adult females on whom we had the most data available at each site (Tuanan: 4098–5161 h, $\overline{\mathrm{X}}$ = 4644 h, Suaq: 320–1168 h, $\overline{\mathrm{X}}$ = 679). We determined the number of processing steps of all the food items that together make up 90 % of their diets (in terms of feeding time). Processing steps and examples are described in detail in Table 1.

### Data analysis

All analyses and plots were done using R [59]. Data were analyzed using general linear mixed models (GLMM), using lmer as implemented in the package lme4 [60], with individual included as a random factor, to account for the fact that data were collected on the same individuals at multiple times, at different stages of their development. Also, in the analysis of the feeding rates, the food item was included as random factor since the same food items occurred multiple times in the data set (eaten by different immatures at different ages). Statistical significance of the fixed effects was assessed using cftest of the multcomp package [61]. Models and their factors were selected based on the Akaike information criteron (AIC). In the case of diet repertoires and feeding rates the best description of age effects was sigmoid, for which we used the pracma package [62].

For the figures, nonlinear relationships in the data were drawn after the respective general linear model (GLM of the stats package, with age as a sigmoid factor), excluding the random factor (individual and species).

## Results

### Testing the needing-to-learn hypothesis

#### What to eat: food selection competence

**Fig. 2:**
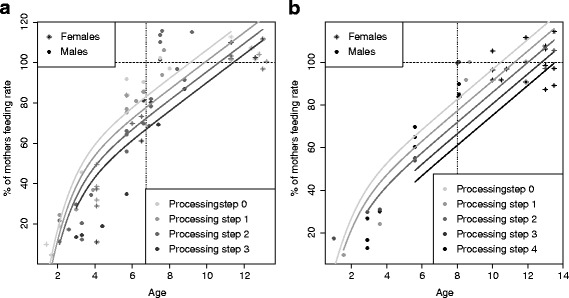
Development of feeding rates: Immatures’ feeding rates, expressed as percentage of their mother’s feeding rates, as a function of age and processing complexity of the food items, at Tuanan (**a**) and Suaq (**b**). The vertical dotted line shows mean weaning age at each population. The horizontal dashed line marks adult-level feeding rate (100 %)

To assess the development of food selection competence, we examined the immatures’ diet composition as a function of age. We found that with increasing age, immatures ate an increasing number of food items, and at the age of weaning they reached a repertoire size that is between 80 and 99 % of their mothers’ repertoire size. When examining sex differences, we found a significant interaction effect between age and sex, implying that female immatures attained a broader diet earlier than their male peers, who by the age of weaning seemed to reach only 80 % of their mother’s diet repertoire size (Table 2, Fig. 2).

**Table 2:** GLMM with diet repertoire size in percentage of the mother’s diet as a dependent variable: Effects, estimates, standard errors and p-values as well as number of levels for the categorical variables

Effect	Type of effect	Estimate	Std-Error	*P*-value	N (31)
Age	Fixed	6.84	1.17	<**0.001**	cont.
*sigmoid* (Age)	Fixed	119.38	9.32	<**0.001**	-
Sex (male = 0)	Fixed	5.26	6.35	0.408	2
Age x Sex (male = 0)	Fixed	−2.67	0.93	**0.004**	-
Follow hours	Fixed	0.001	0.002	0.46	cont.
Individual	Random	-	-	-	6

The number in parentheses represents the total number of age individual means

Bold font indicates significance at the 0.05 leve﻿l

#### How to eat: food processing competence

**Fig. 3:**
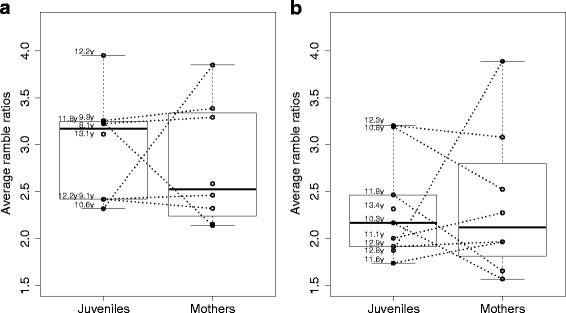
Development of Ramble ratios: Average daily ramble ratios for independently ranging immatues (juveniles) and mothers at Tuanan (**a**) and Suaq (**b**). The numbers next to the juvenile data points indicate ages (in years). The juvenile points are always paired with a data point of their mother collected in the same 5 month period (in 3 cases this data was not available)

In terms of food processing competence, we tested the effects of age, food processing complexity, sex and site on feeding rates simultaneously, using GLMM. We found that feeding rates increased with age. For the more difficult to process food items, adult-level feeding rates were reached later relative to easier to process items (Table 3a, Fig. 3). Also, immatures started feeding on more difficult to process food items later in their development compared to easier items (Fig. 3), in line with the notion that feeding techniques are learned rather than intrinsic [51, 52].

**Table 3:** GLMMs of feeding rate, expressed as percentage of the mothers’ feeding rates, as a dependent variable, with (a) and without (b) including the number of processing steps as an independent variable

Effect	Effect type	Estimate	Std-Error	P-value	N (128)	AIC
a)						
Age	Fixed	7.96	0.94	<**0.001**	cont.	964
*sigmoid*(Age)	Fixed	−36.34	56.24	0.52	-	
Processing steps	Fixed	−4.77	1	<**0.001**	5	
Sex (male = 0)	Fixed	−19.58	10.5	0.062	2	
Site (Tuanan = 0)	Fixed	−120.91	70.31	0.085	2	
Age x Sex	Fixed	4.94	1.27	<**0.001**	-	
*sigmoid*(Age) x Site	Fixed	126.58	71.67	0.078	-	
Individual	Random	-	-	-	21	
Food Item	Random	-	-	-	34	
b)						
Age	Fixed	7.88	0.9	<**0.001**	cont.	981
*sigmoid*(Age)	Fixed	−69.13	55.47	0.212	-	
Sex (male)	Fixed	−21.27	10.09	**0.035**	2	
Site (Tuanan = 0)	Fixed	−130.81	69.44	0.06	2	
Age x Sex	Fixed	5.56	1.24	<**0.001**	-	
*sigmoid*(Age) x Site	Fixed	139.4	70.88	**0.049**	-	
Individual	Random				21	
Food Item	Random				34	

Shown are effects, estimates, standard errors and p-values as well as number of levels for the categorical variables and AIC values of the models. The number in parentheses represents the total number of individual - age - species combinations

Bold font indicates significance at the 0.05 leve﻿l

At both sites, adult-level feeding rates for all processing classes were reached after weaning but well before age at first reproduction (Fig. 3). In relation to absolute age, Suaq immatures reached adult-like feeding rates later than their Bornean peers (Fig. 3). There was a trend for a site difference in the development of feeding rates, as indicated by a trend for an interaction of age with site (sigmoid(age) x site, Table 3a). If processing steps were excluded from the model, the site difference, as well as the interaction term of age with site (sigmoid(age) x site, Table 3b) became significant, which showed that without correcting for food complexity, Suaq immatures developed adult-level feeding rates later than Tuanan immatures (Table 3b). Including processing steps significantly improved the overall fit of the model, showing that processing complexity of the food explained a significant proportion of the variance in our data (Table 3a + b, model comparison: Chi^2^ = 97.6, *p* < 0.001).

After weaning, at both sites, but more so at Tuanan, feeding rates of independent immatures exceeded those of their mothers, especially for easier to process items (Fig. 3). Interestingly, at both sites males acheived higher feeding rates faster compared to same-aged females at both sites (Table 3a, Fig. 3).

To assess whether in general, slower feeding rates of the immature individuals could be a consequence of smaller body size or the lack of strength, we controlled for the effects of fruit size (average length) and the toughness of the fruits (see [63]). Including toughness and fruit size as factors led to an improvement in the overall fit of the model but the effects themselves were not significant (Additional file 1: Table S4).

#### When and where to eat: food locating competence

**Fig. 4:**
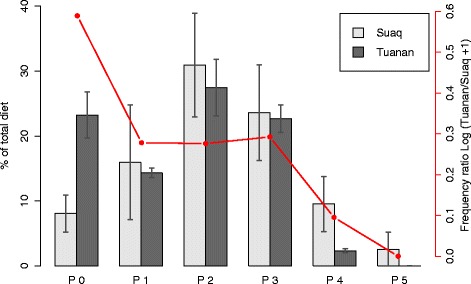
Body size development: Measures of whole arm, fore arm and upper arm versus age of the Suaq immatures in absolute values (**a**) and in percentage of the average adult females (**b**). Forearm length versus age of the immatures at Tuanan and Suaq in absolute values (**c**) and in percentage of the average adult females (**d**). The Tuanan data were retrieved from [54]

Regarding competence in locating food sources and exploiting them efficiently, we found that ramble ratios of the travel routes of the independently ranging immatures did not differ from those of their mothers: Average daily ramble ratios of the adult females ranged from 1.6 – 3.9 at Tuanan and 2.1 – 3.8 at Suaq, with standard deviations across the different days of the same individual ranging from 0.3 – 1.5 at Tuanan and 0.5 to 2.2 at Suaq. Independent immatures showed average daily ramble ratios ranging from 1.7 - 3.2 at Tuanan and 2.1 – 4 at Suaq, with standard deviations ranging from 0.6 - 2.8 at Tuanan and 0.5 - 1.9 at Suaq. Thus, there was no evidence that independently ranging immatures showed higher or more variable ramble ratios in their travel routes than adults (Fig. 4 and Additional file 2: Figure S2, Table 4 a). However, there was a significant difference in ramble ratios between the two sites with Tuanan individuals showing lower values and thus more direct travel routes than individuals at Suaq (Fig. 4, Table 4a). We found no age effect on the average daily ramble ratios among the independently ranging immatures (Fig. 4, Table 4b): even the youngest and thus newly independently ranging immatures showed ramble ratios in the range of adult females. We also found no difference between mothers and immatures using a paired design in which we compared each independently ranging immature with its mother (Fig. 4).

**Table 4:** GLMM with average daily ramble ratio of the mothers and independent immatures as dependent variable, age class and site as fixed effect as well as individual as random effect

Nr.	Effect	Effect type	Estimate	Std-Error	*P*-value	N
a	Age class (mother = 0)	Fixed	−0.1	0.25	0.672	2
	Site (Tuanan = 0)	Fixed	−0.52	0.25	**0.035**	2
	Individual	Random	-	-	-	15 (32)
b	Age	Fixed	0.02	0.1	0.863	cont.
	Site (Tuanan = 0)	Fixed	−0.69	0.3	**0.021**	2
	Individual	Random	-	-	-	8 (17)

Shown are estimates, standard errors, *p*- values and number of levels for the categorical variables (a). GLMM with average daily ramble ratios of the independently ranging immatures animals as dependent variable, age and site as fixed effects as well as individual as random effect. Estimates, standard errors, p- values and number of levels for the categorical variables (b). The numbers in parentheses indicate the total number of age individual means

Bold font indicates significance at the 0.05 leve﻿l

#### Dietary complexity of the two sites

**Fig. 5:**
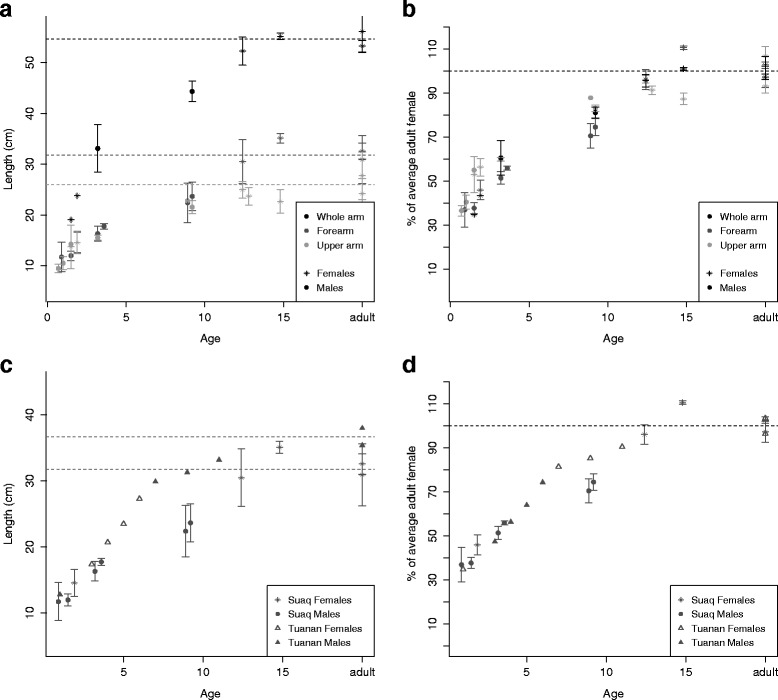
Dietary complexity: Distribution of the different processing steps in percent of the total diet (for the food items that form the top 90 % of the total diet) of 4 adult females at Tuanan and Suaq each. The red line indicates the site frequency ratio of each given processing step (log (frequeny at Tuanan/frequency at Suaq +1))

In terms of dietary complexity, we found that the two study sites showed different distributions of the frequencies of the different processing steps (Fig. 5). Whereas at Tuanan individuals ate a higher share of items that can be ingested without any processing (steps = 0) than Suaq individuals, they ate a lower number of items with 4 processing steps relative to Suaq individuals. Moreover, processing requiring 5 steps (tool use) only occurs at Suaq. Frequencies of processing steps 1–3 were very similar for the two sites. Overall, therefore, the relative frequency of a given processing step at Tuanan (its value divided by the corresponding value at Suaq) decreased with increasing processing complexity (Spearman: r = −0.83, *p* = 0.058, *n* = 6). The diet at Suaq is therefore more complex than the diet at Tuanan.

### Testing the expensive brain framework

#### Body growth

Preliminary data on body growth suggest that immatures grow until around the age at female first reproduction (Fig. 1a + b), even though males are known to continue growing after this age. We discerned a trend for Suaq immatures to show smaller body sizes in absolute values as well as relative to the adult female body size than their Tuanan peers after weaning (Fig. 1 c + d). At the current state, these data are insufficient for any detailed statistics.

## Discussion

### Needing to learn or needing to grow?

To investigate whether the late age at first reproduction and the late age of weaning in orangutans is best explained by the time needed to acquire skills (needing-to-learn hypothesis) or rather by tradeoffs in energy allocations to brain and body (expensive brain frame work), we examined foraging skill development and body growth trajectories of immatures of two populations of wild orangutans. Our results showed that adult like diet repertoires (a measure of food selection competence) are reached around the age of weaning. Adult like feeding rates (a measure of food processing competence) are attained shortly thereafter and adult like ramble ratios (a measure of food locating competence) are at adult levels right at the start of ranging independence, soon after weaning. Thus, in orangutans, the acquisition of foraging skills does not impose a direct limit on age at first reproduction, as proposed by the classic version of the needing-to-learn hypothesis. On the other hand, body size measurements showed that immatures continue to grow until female age at first reproduction. This suggests that as predicted by the expensive brain framework, the need to reach adult body size might determine the length of the developmental period in orangutans.

#### Evidence for an altered version of the needing-to-learn hypothesis

Although reaching adult-level feeding skills doesn’t directly limit the onset of reproduction, we found evidence that foraging skills might play a crucial role for the timing of weaning. In contrast to all other gregarious primates, by the age of weaning, immature orangutans have to be fully self-sufficient: in most populations weaned immatures increasingly range alone and thus can’t rely on social information and are fully dependent on their own knowledge and skills [37, 44]. Weaned immatures must have reached sufficient skill levels to support their small but growing body (see below). The development of the diet repertoire showed that food selection competence is reached around the age of weaning, but that adult-level processing of these foods is achieved later, especially for food items requiring more processing steps. Ramble ratios did not differ between adults and independently ranging immatures. Although these may also be influenced by factors other than food locating competence, such as avoiding or associating with other individuals, the absence of any systematic difference between age classes suggests that independently ranging immatures from the beginning of independence have no difficulty to locate food sources in the forest. This is consistent with the results of an earlier study showing that shortly before the age of weaning, immatures at Tuanan seem to recognize at least 50 % of their food patches before their mothers [64]. It thus seems that the critical skills for dietary independence in orangutans are competence in food selection and food processing, rather than in food locating.

The diets of adult female orangutans at Tuanan comprise of more than 170 food items of more than 110 different plant species (Additional file 2: Figure S1). These numbers are very comparable to findings of long-term studies on wild chimpanzees (e.g. [65, 66]). However, for the adult females at Tuanan, even after 4000 observation hours, collected over multiple years, new items are still added to the diet (Additional file 2: Figure S1), suggesting there is no such thing as a true diet. This finding also reflects the fluctuating food availability in most orangutan habitats and the lack of consistent seasonal patterns in fruiting [46]. Thus, it is plausible that it takes an immature orangutan multiple years to witness all the items in their mothers’ diets and probably even more time to memorize them. In general, juvenile animals are very vulnerable to food shortages ([12], see below). A broad diet is crucial to buffer episodes of food scarcity in a habitat with fluctuating food availability. It is unlikely that the incomplete diet repertoires of the immatures are purely an artifact of insufficient processing competence: even though we found that immatures start to feed on more difficult to process items later relative to easy ones, even items of the highest processing classes (including the ones that require tool use) are eaten by the age of 6 years, albeit at a much lower rate [67]. Because toughness of the food item does not affect feeding rates (see Additional file 1: Table S5) and the diets of adults and immatures do not differ in their physical properties [53], it is unlikely that strength differences can explain age patterns in feeding rates. However, feeding rates on the more difficult items, but not on the easy ones, increase after weaning, which suggests that learning continues during juvenility. It thus appears that the age of dietary independence is determined by the time it takes to acquire broad enough repertoires and to reach high enough feeding rates to ensure sufficient energy intake during the next phase of immature development.

The developmental trajectories of the feeding rates showed that from a certain age on, independent immatures feed faster than adult females on easier to process food items. In a study on long-tailed macaques (*Macaca fascicularis*), it was found that larger body size allows for faster processing of large food items [68]. If gape size in relation to food item size determines ingestion rates, a smaller body size may entail advantages for processing smaller items. At weaning, immatures are still significantly smaller than adult females. The smaller body size may explain their higher picking rates for small items for which they reached proficiency earlier. Indeed, all of the items in which immatures exceeded the feeding rates of adult females were flowers and small to medium sized fruits.

As they reach foraging skills around the age of weaning, orangutans follow the classic primate pattern of skill acquisition in relation to life history [11]. Interestingly however, orangutans seem to attain their foraging skills several years later than chimpanzees and gorillas [69–71]. Diet repertoire sizes of wild chimpanzees are highly comparable to the ones of wild orangutans [65, 66] and obvious systematic differences in the skill intensity of their diets are also unlikely. It is possible that the age difference in reaching foraging competence is just a side effect of the overall faster development of chimpanzees and gorillas. However, an equally plausible suggestion is that because orangutans cannot rely on social inputs after weaning they have to reach higher levels of competence before they can be weaned.

All in all, in orangutans, the acquisition of foraging skills does not impose a direct limit on age at first reproduction, as proposed by the classic version of the needing-to-learn hypothesis and competence in knowing what, how, and where to eat is reached long before the age at female first reproduction.

#### Evidence for the expensive brain hypothesis

Growth trajectories obtained by laser photogrammetry showed that by the age of weaning, immature orangutans have only reached around 75 % of adult female body size in terms of arm length, which probably corresponds to around 50 % of adult volume and weight. Interestingly, this % of adult size at weaning seems highly comparable for immatures at Suaq and Tuanan (Additional file 2: Figure S3). Apart from substantial body growth, there is also considerable brain development during the juvenile period: even though the great ape brain is fully grown by the age of weaning [21, 72], it undergoes a period of synaptic remodeling (overproduction of axons and synapses followed by rapid pruning) and restructuring during juvenility and adolescence [73–76]. Thus, the energetic needs during juvenility imposed by body growth and brain fine-tuning are high [18]. However, constant energy flow to the developing brain is vital since it cannot adjust to temporary energy shortages [16, 19]. Brain starvation causes permanent cognitive impairments and thus reduced adult performance later in life [77, 78]. The optimal body size at the age of weaning and the conservative growth likely act to minimize the risk of starvation but do so at the expense of a longer juvenile period and delayed reproduction [12].

#### Evidence for a combination of needing-to-learn and expensive brain

One option that has not yet been discussed in the literature is that the two limiting factors of the two hypotheses are competing with each other and are thus limiting at the same time. It might be that energetic investment into learning implies a reduced investment into body growth and vice versa. Also, given that the brain is the structure that allows for learning developing, the brain must be tightly linked to learning rates. Accordingly, in humans it has been shown that brain differentiation during childhood requires peak levels of brain glucose uptake and happens at the expense of body growth [22]. Here and in previous studies we show that immature orangutans learn most of their skills during infancy [51, 52]. It might thus be that during this learning intense period, energy is primarily used for learning to reach sufficient skill levels by the time of nutritional independence. During juvenility, when skills are more or less in place, the body then has to catch up before reproduction can start. This scenario is in line with the species difference in weaning age: in a population where there is less to learn, adult skill levels can be reached faster and thus nutritional independence is reached earlier. The deducted growth trajectories suggest that after weaning the immatures at Suaq probably show a slower growth than their Bornean peers. This preliminary finding further supports the argument that growth competes with learning: since skills are generally reached later at Suaq, it is likely that also during the juvenile period the investment in fine tuning and perfecting these skills is higher. The most extreme example for this is tool use, where proficiency is reached only during late juvenility or even adulthood [67]. A higher energetic investment into continued learning during the unprovisioned juvenile period could explain the difference in growth rate during this period.

Thus, even though in the end body size appears to be directly limiting on age at first reproduction, the connection between body growth and skill learning might be more interactive than previously assumed. Consequently the classic needing-to-learn hypothesis as well as classic life history might have to be modified into a combined approach, which takes a potential interdependence of growth and learning into account.

### Sex differences

We found unexpected sex differences in the development of diet repertoires and feeding rates. Whereas female immatures develop broader diets faster than males, male immatures reach faster feeding rates earlier than females. In adult orangutans, males in general spend less time feeding than females [53, 79, 80]. Sex differences in various aspects of adult foraging have been found in several other primate species (e.g. [81, 82]). Those differences have been ascribed to either differing energetic needs associated with sexual size dimorphism or the costs of female reproduction [82–85]. Immature orangutan males will, after going through a stage when they are unflanged and at least initially well within the range of female sizes, eventually grow into flanged males, which are much bigger than adult females [86]. However, recent findings show that in orangutans, there is remarkably little variation in total daily energy intake across age sex classes [80].

As in all mammals, orangutan females have to be able to sustain pregnancy and lactation. Reproducing females increase their food intake relative to basic need by only about 25 % [85]. During pregnancy, females are probably more dependent on high food quality, in terms of nutrient composition and plant allelochemicals. Also, reproducing orangutan females likely have more stable energy intakes compared males because their nutritional state directly influences the development of her offspring, for which energy shortages are detrimental (see below). Thus, female diets are likely to be broader and thus more balanced and less susceptible to fluctuations of food availability.

It has been proposed that, depending on the underlying mechanisms, sex differences in foraging are closely correlated with female reproductive status [83] or emerge parallel to the developmental onset sexual size dimorphism [82, 84, 87]. Our findings of sex differences in diet composition and feeding rates among juvenile orangutans are not in line with these predictions. Furthermore, just as in orangutans, immature ring-tailed lemurs (*Lemur catta*; [82]), long-tailed macaques [88] and tufted capuchin monkeys (*Cebus nigritus*: [81]) are characterized by sex differences in several aspects of feeding that match those of adults. Also, immature female chimpanzees acquire proficiency in termite fishing tool use earlier, spend more time watching others termite-fish than their male peers [84], whereas greater female proficiency in tool use has been reported for captive bonobos [89]. The most parsimonious alternative hypothesis for many species, namely that sex differences are caused by resource partitioning [82, 90], is unlikely to apply in the chimpanzee and bonobo examples and can be ruled out in the largely solitary Bornean orangutan (see also [42]). In conclusion, in species where repertoires and techniques are learned rather than intrinsic, differences caused by the balance between reproductive load and sexual dimorphism in adult size might already emerge during the juvenile period, suggesting that female and male immatures prepare for sex specific adult niches at this stage of development. In general, to determine the biological significance of these sex differences in immature orangutan diet repertoires and feeding rates, one would have to look in more detail at sex differences in adults.

### Between-site differences

We found that immatures at Tuanan may possibly grow faster than at Suaq, which could be linked to their earlier weaning and a lower overall complexity of their diet. Since the toughness of the food item did not affect feeding rates (see Additional file 1: Table S5) and immature and adult diets are similar in toughness [53], it is unlikely that differences in feeding rates are due to the smaller body size and thus reduced strength of the immatures. Interestingly, when we controlled for the complexity of the food items by including it as a factor in the model, the difference in age of skill competence (i.e. how fast feeding rates increase as a function of age) between Suaq and Tuanan became non-significant (Fig. 3, Table 3 a + b). Thus, it seems as if skills of equal complexity develop at a very similar pace at both populations, but that due to the higher complexity of their diet, Suaq immatures have more to learn, so weaning gets delayed. Immatures in both populations are weaned at the same level of feeding skills relative to the overall skill level of the population: by the age of weaning, adult feeding rates are reached for the easier items whereas for the more complex items, adult-like feeding rates are reached after around two thirds of the juvenile period. The fact that even after controlling for their higher complexity level, there remains a trend that Suaq individuals reach adult-like feeding rates later than Tuanan individuals (i.e. a trend for a positive interaction between site and age on skill competence; see Table 3 a) could be the result of investments in acquiring more complex skills, which reduce the amount of time and energy available to learn the more basic skills and slow down the learning process of those.

Only at Suaq individuals show tool use, the most complex class of food processing. Competence in tool use is difficult to quantify but there is evidence that orangutans reach full competence in tool use only during late juvenility or even early adulthood [67]. However, since not all populations of Sumatran orangutans use tools, it is unlikely to be essential for an individual’s survival. Nonetheless, even if we ignore tool use, the overall complexity of the diet is higher at Suaq than at Tuanan. The near absence of processing step 4 at Tuanan suggests that the probability to develop a highly complex technique, such as tool use, is higher in populations where techniques with long chains of functionally dependent actions are more prevalent.

This pattern suggests there has been correlated evolution between a population’s dietary complexity, somatic growth, and weaning age. This correlation could equally reflect the need to reach greater dietary complexity, which then selected for later weaning, or later weaning being caused by slower life-history pace, which then provided the extended learning period that made the evolution of a greater dietary complexity possible. The fact that the overall level of a population’s dietary complexity affects the pace of immature development implies that it is important for individuals within a given population to acquire the full range of skills. However, top-end complexity skills seem to only develop and be maintained in a population under certain preconditions, such as a prolonged mother offspring association or in general increased opportunities for social learning. Perhaps after weaning, juveniles are too busy surviving to focus on learning totally new skills. More generally, this might also apply to the overall complexity level of the diets of different orangutan populations. Our findings are therefore consistent with the finding that there is a correlation between the complexity of the diet niche and (i) the pace of development, as found in primates, and (ii) the relative length of food provisioning, as found in carnivores [91]. More specifically, applied across mammal species, our results suggest a correlation between weaning age in relation to the age at first reproduction and the complexity of the diet as well as body size at the age of weaning.

## Conclusions

We could show that in orangutans, both the need to learn foraging skills and energetic constraints have severe impacts on life history. Whereas the age at first reproduction seems to be primarily determined by competing energetic investments in brain and body, the age at weaning seems to be connected to how fast foraging skills are acquired. However, it is likely that energetic investment in learning competes with the development of brain and body such as that growth can only be completed after the intense learning period of infancy. Accordingly, both factors may be limiting overall development at the same time. In populations with a higher dietary complexity, immatures have more to learn and thus reach adult skill levels later. At the same time, they seem to grow more slowly and are weaned later than immatures at populations with lower dietary complexity, where the minimum skill set to survive seems to be reached earlier. Sex differences in the development of foraging skills suggest that immature female and male orangutans prepare for sex specific adult niches. Thus, the overall adult niche (in terms of the population’s overall complexity level of foraging techniques as well as in terms of sex specific foraging complexity) has a significant impact on immature skill development. Applied across different primate species, this implies that weaning should be reached later when there is more to learn.

## Additional files

Additional file 1: Table S1. Overview of the data used for the different analyses. Study site, focal individual, sex and date of birth of the focal individual, mother of the focal individual, as well as the years in which the different types of data (diet data, feeding rates, ramble ratios and laser measurements) were taken. **Table S2.** GLMM with absolute feeding rate of the adult females as dependent variable. To see if the if there is a difference between feeding rates obtained by video coding as opposed to direct observation this was included as as a binary variable (“Video”). Shown are effects, estimates, standard errors and p-values as well as number of levels for the categorical variables. The number in parentheses represents the total number of individual feeding rates included in the analyses: Only rates of fruits on which we had data from the same individual obtained via video coding as well as via direct observation were included. **Table S3.** GLMM with feeding rate in percentage of the mothers feeding rates as a dependent variable. To see if the presence absence of a simultaneous feeding rate taken on the mother has an effect it was included as a binary variable (“Simultaneous mother sample”). Effects, estimates, standard errors and p-values as well as number of number of levels for the categorical variables and AIC values of the models. The number in parentheses represents the total number of individual - age - species combinations.  **Table S4.** GLMM with feeding rate of the Tuanan immatures in percentage of the mothers feeding rates as a dependent variable without (a) and with fruit toughness and size included as a fixed effect (b). Effects, estimates, standard errors and p-values as well as number of levels for the categorical variables and AIC values of the model. (ZIP 23 kb)
Additional file 2: Figure S1. Diet repertoire size in relation to follow effort: The number of recorded food items versus the number of hours of follow data collected for the three adult females with the most data available at Tuanan. 500 follow hours correspond to roughly 1 year ($\overline{\mathrm{X}}$ = 13.1 months), the overall observation time was 9.5 years. The total number of plant species recorded for each of these females was 109 – 113 ($\overline{\mathrm{X}}$ =110.3). For insects we only counted termites, ants, bees (honey) and caterpillars as food items and did not distinguish between the different species. Thus, the number of non-plant food items was 4 for each adult female. **Figure S2.** Development of ramble ratios over age: Average daily ramble ratios versus age for immatures at Suaq and Tuanan. **Figure S3.** Body size comparisons: Estimated growth trajectories bases on forearm measurements for immatures at Suaq and Tuanan. The solid lines were attained via smoothing functions in R (smooth.spline in the stats package). The horizontal lines show average weaning ages at both populations. The Tuanan data were retrieved from [54]. (DOCX 36 kb)
