# Correction to: development of foraging skills in two orangutan populations: needing to learn or needing to grow?

**DOI:** 10.1186/s12983-017-0237-6

**Published:** 2018-02-08

**Authors:** Caroline Schuppli, Sofia I. F. Forss, Ellen J. M. Meulman, Nicole Zweifel, Kevin C. Lee, Evasari Rukmana, Erin R. Vogel, Maria A. van Noordwijk, Carel P. van Schaik

**Affiliations:** 1 Department of Anthropology, University of Zürich, Winterthurerstrasse 190, 8057 Zürich, Switzerland; 2 Fakultas Biologi, Universitas Nasional, Jl. Sawo Manila, RT.14/RW.3, Ps. Minggu, DKI, Jakarta, Indonesia; 3 Department of Anthropology, Rutgers, The State University of New Jersey, New Brunswick, NJ 08904 USA

## Correction

Upon publication of this article [1] it was noticed corrections were not carried out correctly and subsequently figure placement and some figure citations were incorrect.

The original article has now been updated to reflect the correct figure placement and their respective citations within the text and the publisher apologizes for any inconvenience caused.
