# Supplementary figures and images for: Development of foraging skills in two orangutan populations: needing to learn or needing to grow?
Source: Front Zool. 2016 Sep 29;13:43. doi: 10.1186/s12983-016-0178-5 (PMC5041519; doi:10.1186/s12983-016-0178-5)

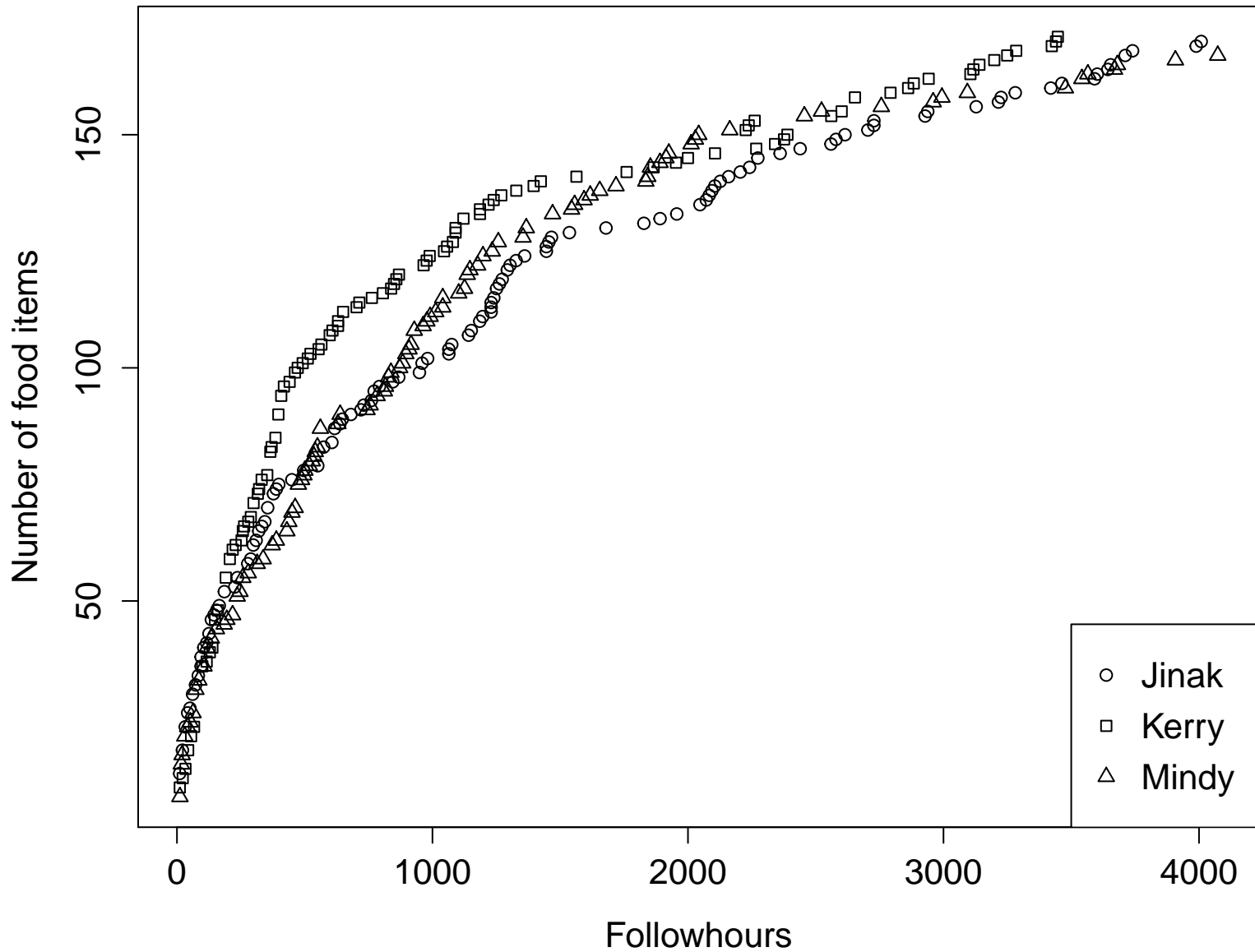

Supplement: Additional file 1: Table S1. — Overview of the data used for the different analyses. Study site, focal individual, sex and date of birth of the focal individual, mother of the focal individual, as well as the years in which the different types of data (diet data, feeding rates, ramble ratios and laser measurements) were taken. Table S2. GLMM with absolute feeding rate of the adult females as dependent variable. To see if the if there is a difference between feeding rates obtained by video coding as opposed to direct observation this was included as as a binary variable (“Video”). Shown are effects, estimates, standard errors and p-values as well as number of levels for the categorical variables. The number in parentheses represents the total number of individual feeding rates included in the analyses: Only rates of fruits on which we had data from the same individual obtained via video coding as well as via direct observation were included. Table S3. GLMM with feeding rate in percentage of the mothers feeding rates as a dependent variable. To see if the presence absence of a simultaneous feeding rate taken on the mother has an effect it was included as a binary variable (“Simultaneous mother sample”). Effects, estimates, standard errors and p-values as well as number of number of levels for the categorical variables and AIC values of the models. The number in parentheses represents the total number of individual - age - species combinations. Table S4. GLMM with feeding rate of the Tuanan immatures in percentage of the mothers feeding rates as a dependent variable without (a) and with fruit toughness and size included as a fixed effect (b). Effects, estimates, standard errors and p-values as well as number of levels for the categorical variables and AIC values of the model. (ZIP 23 kb) [file 12983_2016_178_MOESM1_ESM.zip › Additional file 2/Figure_S1.pdf]

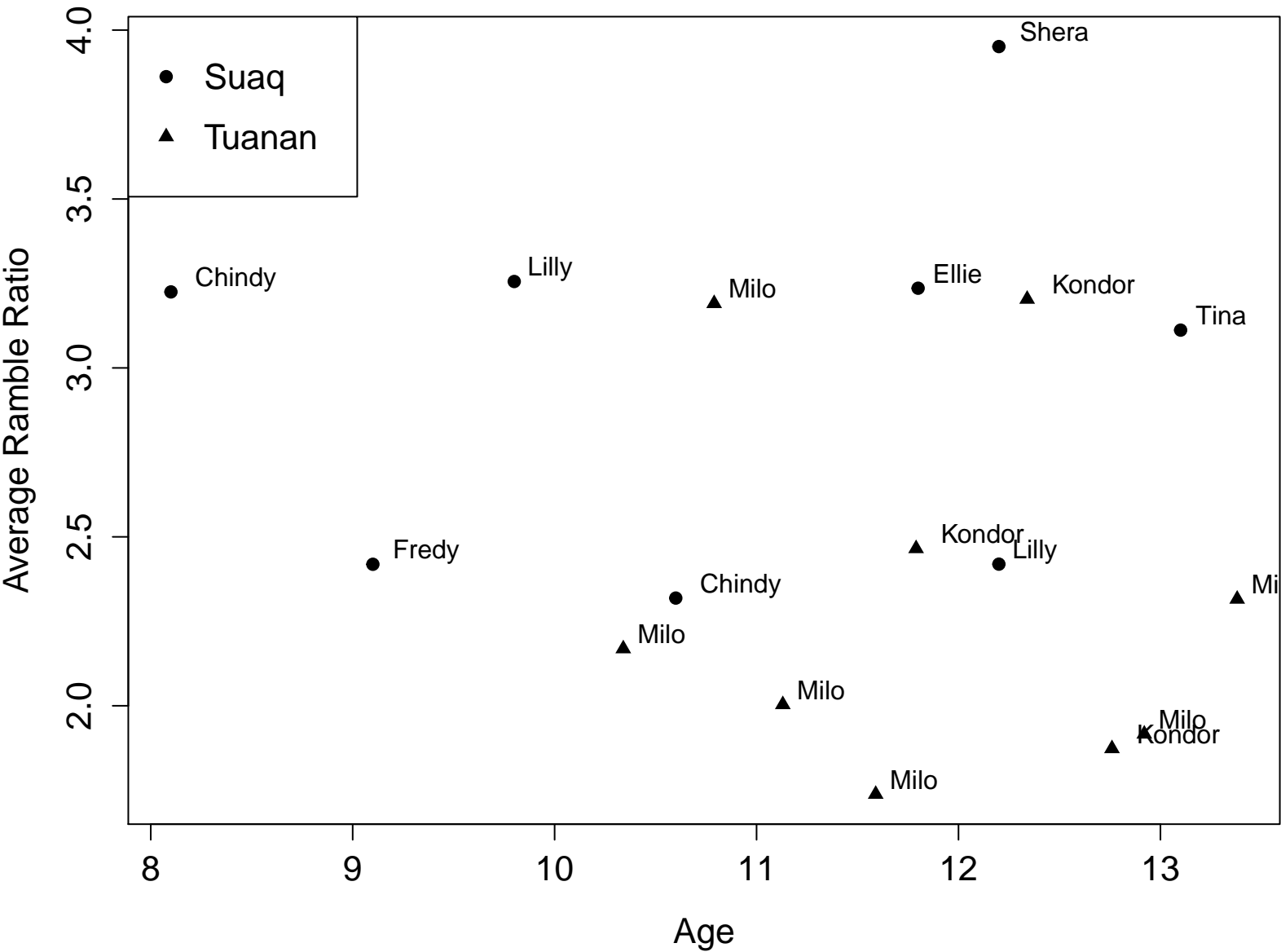

Supplement: Additional file 1: Table S1. — Overview of the data used for the different analyses. Study site, focal individual, sex and date of birth of the focal individual, mother of the focal individual, as well as the years in which the different types of data (diet data, feeding rates, ramble ratios and laser measurements) were taken. Table S2. GLMM with absolute feeding rate of the adult females as dependent variable. To see if the if there is a difference between feeding rates obtained by video coding as opposed to direct observation this was included as as a binary variable (“Video”). Shown are effects, estimates, standard errors and p-values as well as number of levels for the categorical variables. The number in parentheses represents the total number of individual feeding rates included in the analyses: Only rates of fruits on which we had data from the same individual obtained via video coding as well as via direct observation were included. Table S3. GLMM with feeding rate in percentage of the mothers feeding rates as a dependent variable. To see if the presence absence of a simultaneous feeding rate taken on the mother has an effect it was included as a binary variable (“Simultaneous mother sample”). Effects, estimates, standard errors and p-values as well as number of number of levels for the categorical variables and AIC values of the models. The number in parentheses represents the total number of individual - age - species combinations. Table S4. GLMM with feeding rate of the Tuanan immatures in percentage of the mothers feeding rates as a dependent variable without (a) and with fruit toughness and size included as a fixed effect (b). Effects, estimates, standard errors and p-values as well as number of levels for the categorical variables and AIC values of the model. (ZIP 23 kb) [file 12983_2016_178_MOESM1_ESM.zip › Additional file 2/Figure_S2.pdf]

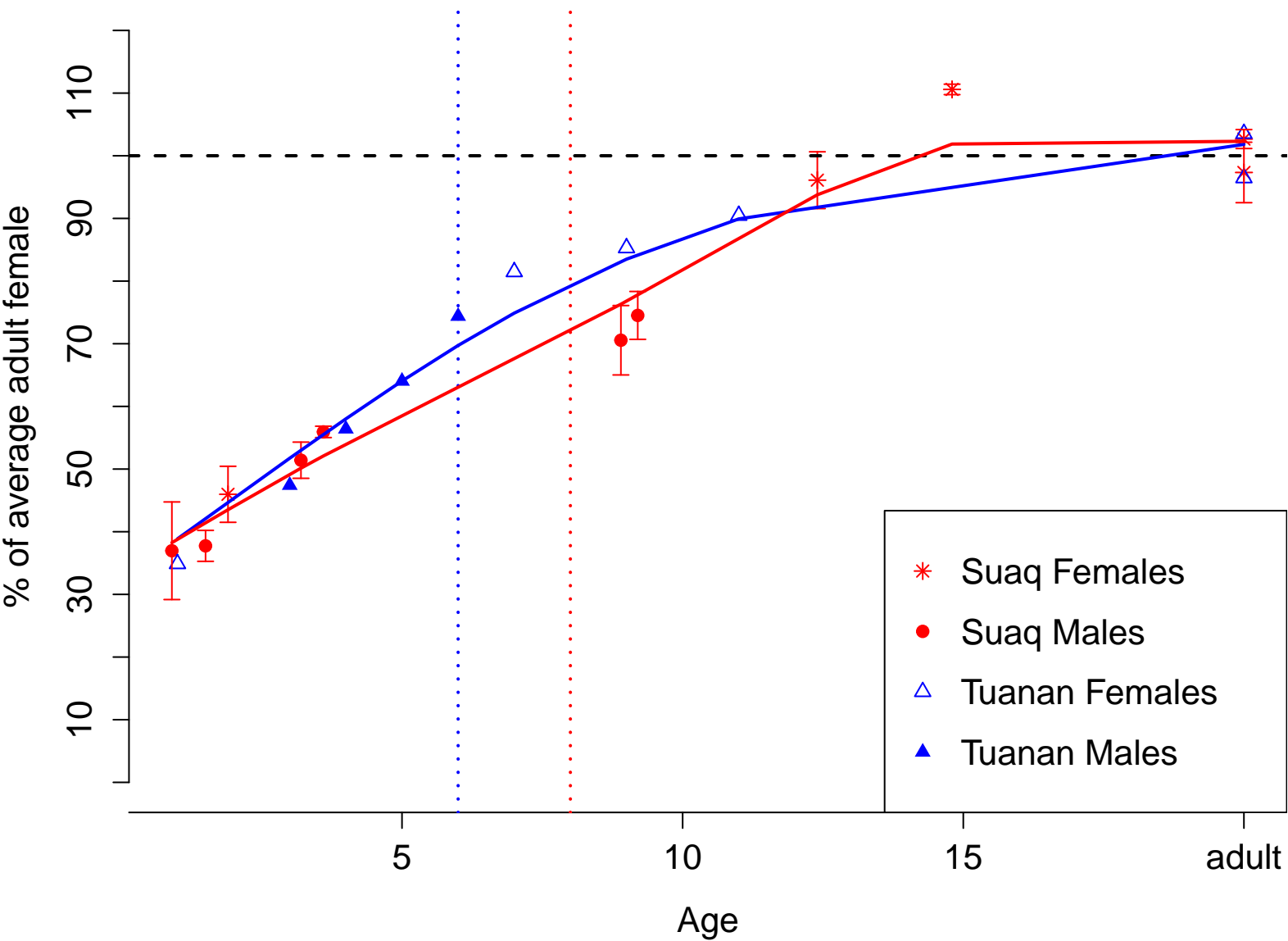

Supplement: Additional file 1: Table S1. — Overview of the data used for the different analyses. Study site, focal individual, sex and date of birth of the focal individual, mother of the focal individual, as well as the years in which the different types of data (diet data, feeding rates, ramble ratios and laser measurements) were taken. Table S2. GLMM with absolute feeding rate of the adult females as dependent variable. To see if the if there is a difference between feeding rates obtained by video coding as opposed to direct observation this was included as as a binary variable (“Video”). Shown are effects, estimates, standard errors and p-values as well as number of levels for the categorical variables. The number in parentheses represents the total number of individual feeding rates included in the analyses: Only rates of fruits on which we had data from the same individual obtained via video coding as well as via direct observation were included. Table S3. GLMM with feeding rate in percentage of the mothers feeding rates as a dependent variable. To see if the presence absence of a simultaneous feeding rate taken on the mother has an effect it was included as a binary variable (“Simultaneous mother sample”). Effects, estimates, standard errors and p-values as well as number of number of levels for the categorical variables and AIC values of the models. The number in parentheses represents the total number of individual - age - species combinations. Table S4. GLMM with feeding rate of the Tuanan immatures in percentage of the mothers feeding rates as a dependent variable without (a) and with fruit toughness and size included as a fixed effect (b). Effects, estimates, standard errors and p-values as well as number of levels for the categorical variables and AIC values of the model. (ZIP 23 kb) [file 12983_2016_178_MOESM1_ESM.zip › Additional file 2/Figure_S3.pdf]
