# Supplementary material for: Development of foraging skills in two orangutan populations: needing to learn or needing to grow?
Source: Front Zool. 2016 Sep 29;13:43. doi: 10.1186/s12983-016-0178-5 (PMC5041519; doi:10.1186/s12983-016-0178-5)
Supplement: Additional file 2: Figure S1. — Diet repertoire size in relation to follow effort: The number of recorded food items versus the number of hours of follow data collected for the three adult females with the most data available at Tuanan. 500 follow hours correspond to roughly 1 year ($\overline{\mathrm{X}}$X¯ = 13.1 months), the overall observation time was 9.5 years. The total number of plant species recorded for each of these females was 109 – 113 ($\overline{\mathrm{X}}$X¯ =110.3). For insects we only counted termites, ants, bees (honey) and caterpillars as food items and did not distinguish between the different species. Thus, the number of non-plant food items was 4 for each adult female. Figure S2. Development of ramble ratios over age: Average daily ramble ratios versus age for immatures at Suaq and Tuanan. Figure S3. Body size comparisons: Estimated growth trajectories bases on forearm measurements for immatures at Suaq and Tuanan. The solid lines were attained via smoothing functions in R (smooth.spline in the stats package). The horizontal lines show average weaning ages at both populations. The Tuanan data were retrieved from [54]. (DOCX 36 kb) [file 12983_2016_178_MOESM2_ESM.docx]

**Supplementary material**

**Table S2:** GLMM with absolute feeding rate of the adult females as dependent variable. To see if the if there is a difference between feeding rates obtained by video coding as opposed to direct observation this was included as as a binary variable (“Video”). Shown are effects, estimates, standard errors and p-values as well as number of levels for the categorical variables. The number in parentheses represents the total number of individual feeding rates included in the analyses: Only rates of fruits on which we had data from the same individual obtained via video coding as well as via direct observation were included.

| **Effect** | **Effect type** | **Estimate** | **Std-Error** | **P-value** | **N (27)** |
| --- | --- | --- | --- | --- | --- |
| Video | Fixed | -3.05 | 2.19 | 0.163 | 2 |
| Species | Random | - | - | - | 5 |
| Individual | Random | - | - | - | 4 |

**Table S3.** GLMM with feeding rate in percentage of the mothers feeding rates as a dependent variable. To see if the presence absence of a simultaneous feeding rate taken on the mother has an effect it was included as a binary variable (“Simultaneous mother sample”). Effects, estimates, standard errors and p-values as well as number of number of levels for the categorical variables and AIC values of the models. The number in parentheses represents the total number of individual - age - species combinations.

| **Effect** | **Effect type** | **Estimate** | **Std-Error** | **P-value** | **N (128)** | **AIC** |
| --- | --- | --- | --- | --- | --- | --- |
| Age | Fixed | 7.99 | 0.94 | **<0.001** | cont. | 962 |
| sigmoid(Age) | Fixed | -37.16 | 56.22 | 0.51 | - |  |
| Processing step | Fixed | -4.71 | 1.02 | **<0.001** | 5 |  |
| Sex (male= 0) | Fixed | -20.08 | 10.53 | 0.057 | 2 |  |
| Site (Tuanan= 0) | Fixed | -123.43 | 70.44 | 0.08 | 2 |  |
| Age x Sex | Fixed | 4.94 | 1.27 | **<0.001** | - |  |
| sigmoid(Age) x Site | Fixed | 129.65 | 72.04 | 0.071 | - |  |
| Simultaneous mother sample | Fixed | 1.11 | 2.78 | 0.399 | 2 |  |
| Individual | Random | - | - | - | 21 |  |
| Food Item | Random | - | - | - | 34 |  |

**Table S4.** GLMM with feeding rate of the Tuanan immatures in percentage of the mothers feeding rates as a dependent variable without (a) and with fruit toughness and size included as a fixed effect (b). Effects, estimates, standard errors and p-values as well as number of levels for the categorical variables and AIC values of the model.

| Nr | **Effect** | **Effect type** | **Estimate** | **Std-Error** | **P-value** | **N** | **AIC** |
| --- | --- | --- | --- | --- | --- | --- | --- |
|  | Age | Fixed | 8.48 | 0.99 | **< 0.001** | cont. | 482 |
|  | sigmoid(Age) | Fixed | 97.48 | 60.15 | 0.105 | - |  |
|  | Processing step | Fixed | -7.64 | 1.67 | **< 0.001** | 4 |  |
|  | Sex (male= 0) | Fixed | -16.26 | 9.37 | 0.082 | 2 |  |
|  | Age x Sex | Fixed | 4.91 | 1.33 | **< 0.001** | - |  |
|  | Individual | Random | - | - | - | 11 |  |
|  | Food Item | Random | - | - | - | 19 |  |
|  | Age | Fixed | 7.75 | 1.2 | **< 0.001** | cont. | 369 |
|  | sigmoid(Age) | Fixed | 118.74 | 65.34 | 0.069 | - |  |
|  | Processing step | Fixed | -4.71 | 2.5 | **0.05** | 4 |  |
|  | Sex (male= 0) | Fixed | -17.98 | 10.69 | **0.093** | 2 |  |
|  | Age x Sex | Fixed | 5.27 | 1.45 | **<0.001** | - |  |
|  | Fruit toughness | Fixed | -0.27 | 0.9 | 0.77 | cont. |  |
|  | Fruit length Fixed | | -0.26 | 0.18 | 0.138 | cont. |  |
|  | Individual | Random | - | - | - | 10 |  |
|  | Food Item | Random | - | - | - | 15 |  |

**Table S1.** Overview of the data used for the different analyses. Study site, focal individual, sex and date of birth of the focal individual, mother of the focal individual, as well as the years in which the different types of data (diet data, feeding rates, ramble ratios and laser measurements) were taken. *= Suaq individuals that were born outside the current but during a former study period (1994- 1999). Those individuals were identified via DNA analyses of them or their mothers from samples taken during both study periods. **= Individuals of which the birth year had to be estimated because they were born outside the study period (before research activities started or during the multi-year break of the research activities at Suaq). P= Data collected by Abigale Philips (published in [54]).

| **Site** | **Individual** | **Sex** | **Date of birth** | **Mother** | **Diet composition** | **Feeding rates** | **Ramble ratios** | **Laser measurements** |
| --- | --- | --- | --- | --- | --- | --- | --- | --- |
| Suaq | Tina | Female | Early 1998* | Raffi | na | 2011 | 2011 | na |
| Suaq | Shera | Female | Mid 1998** | Chick | na | 2010/2011 | 2010/2011 | na |
| Suaq | Ellie | Female | Early 1999* | Friska | na | 2010/11 | 2010/11 | 2014 |
| Suaq | Lilly | Female | Early 2001** | Lisa | na | 2011 | 2010/11; 2013 | 2013, 2014 |
| Suaq | Chindy | Female | 2003** | Cissy | na | 2010/11; 2013 | 2010/11; 2013 | na |
| Suaq | Fredy | Male | Early 2005** | Friska | na | 2010/11; 2013 | 2014 | 2014 |
| Suaq | Sazu | Male | Mid 2005** | Sarabi | na | 2014 | na | 2014 |
| Suaq | Ronaldo | Male | Early 2006** | Raffi | na | 2014 | na | na |
| Suaq | Lois | Male | August 2010 | Lisa | na | 2011; 2013; 2014 | na | 2013, 2014 |
| Suaq | Frankie | Male | August 2012 | Friska | na | 2013 | na | 2014 |
| Suaq | Cinnamon | Female | May 2012 | Cissy | na | na | na | 2013, 2014 |
| Suaq | Simba | Male | March 2013 | Sarabi | na | na | na | 2014 |
| Suaq | Rendang | Male | March 2014 | Raffi | na | na | na | 2014 |
| Tuanan | Kondor | Female | Early 1999** | Kerry | 2003- 2007 | 2012 | 2010; 2011 | 2010^P^ |
| Tuanan | Streisel | Female | Mid 2001** | Sidony | na | na | na | 2009^P^ |
| Tuanan | Milo | Female | Mid 2001** | Mindy | 2003- 2008 | 2012; 2013; 2014 | 2011; 2012; 2013; 2014 | 2010^P^ |
| Tuanan | Jerry | Male | June 2003 | Jinak | 2003-2011 | 2012; 2014; 2015 | na | 2009^P^ |
| Tuanan | Deri | Male | July 2004 | Desy | na | 2012 | na | 2009^P^ |
| Tuanan | Jip | Male | February 2006 | Juni | 2006- 2013 | 2012; 2013; 2014; 2015 | na | 2010^P^ |
| Tuanan | Kino | Male | January 2007 | Kerry | 2007- 2013 | 2012; 2013; 2014 | na | 2010^P^ |
| Tuanan | Petzy | Male | January 2008 | Pinky | na | 2012 | na | na |
| Tuanan | Mawas | Female | July 2008 | Mindy | 2008- 2013 | 2012; 2014; 2015 | na | 2009^P^ |
| Tuanan | Danum | Male | July 2010 | Desy | na | 2012; 2013; 2014 | na | na |
| Tuanan | Joya | Male | July 2011 | Jinak | na | 2014; 2015 | na | na |
| Tuanan | Kahiyu | Female | Februrary 2012 | Kondor | na | 2013 | na | na |
| Tuanan | Jane | Female | July 2013 | Juni | na | 2014 | na | na |
